# Supplementary material for: Learning consistent subcellular landmarks to quantify changes in multiplexed protein maps
Source: Nat Methods. 2023 May 29;20(7):1058–69. doi: 10.1038/s41592-023-01894-z (PMC10333128; doi:10.1038/s41592-023-01894-z)
Supplement: Supplementary file 1 — Supplementary Note 1, Supplementary Tables 1–6, Supplementary Figs. 1–7 and References [file 41592_2023_1894_MOESM1_ESM.pdf]

nature methods

Article

https://doi.org/10.1038/s41592-023-01894-z

Learning consistent subcellular landmarks to quantify changes in multiplexed protein maps

In the format provided by the authors and unedited

Supplementary Note 1: Detailed comparison of CAMPA and direct pixel clustering in perturbed 184A1 nuclei. 2
Supplementary Table 1: Antibodies and other reagents. 4
Supplementary Table 2: Number of 184A1 cells removed during data cleanup across all conditions. 11
Supplementary Table 3: Number of 184A1 cells and pixels analysed per condition. 12
Supplementary Table 4: Number of HeLa cells removed during data cleanup across all conditions. 13
Supplementary Table 5: Number of HeLa cells and pixels analysed per condition. 14
Supplementary Table 6: Number of cells and molecular profiles per split used for dataset training. 15
Supplementary Figure 1: Robustness to noise and randomness in subsampling and clustering using the 184A1 dataset. 16
Supplementary Figure 2: Mean intensity changes upon Meayamycin or DMSO treatment. 17
Supplementary Figure 3: Pairwise co-occurrence scores between CSLs. 19
Supplementary Figure 4: Examination of outlier cells excluded in Fig. 4 on the basis of the co-occurrence UMAP. 20
Supplementary Figure 5: Quantification of mean intensity changes of each channel in each CSL in HeLa cells upon *SBF2*-knockdown. 22
Supplementary Figure 6: Effect of size-filtering on CSL counts per cell. 23
Supplementary Figure 7: Example cells showing CSLs before and after size-filtering. 24
References 25

Supplementary Note 1: Detailed comparison of CAMPA and direct pixel clustering in perturbed 184A1 nuclei.

Analysis of multiplexed image datasets at the subcellular scale across conditions has previously been performed using direct pixel clustering[1]. In this case, pixel profiles are clustered across the entire dataset of perturbed and non-perturbed cells to identify the predominant pixel 'types' (previously called multiplexed cellular units - MCUs), corresponding to a certain set of colocalising markers (Extended Data Fig. 6a). Because perturbations result in changes to molecular pixel profiles, the abundance of the identified pixel clusters in each cell (or condition) contains information about both the overall and subcellular changes in protein abundance and colocalization. This vector of cluster abundances can be used as a cellular "phenotype" which can be used to cluster cells according to experimental perturbations[1]. Quantitative comparisons of these direct pixel clusters can then be achieved by determining which clusters change in abundance in the different conditions or cell states (Extended Data Fig. 6b). The average molecular profiles which define the clusters are assumed to be independent of the condition, however the extent to which this is a reasonable approximation depends on the clustering resolution. In summary, direct pixel clustering aims to identify pixel combinations that are unique to different conditions, but whose molecular composition is consistent across conditions.

In contrast to this approach, CAMPA identifies CSLs that are found across conditions (and correspond to specific subcellular structures) but whose molecular composition differs between conditions. Using CAMPA, one can therefore make quantitative comparisons of subcellular molecular changes by comparing the molecular compositions of the same CSLs across conditions, rather than by comparing how the abundances of clusters change (as would be done in the direct clustering approach).

As an example to compare these two different approaches, we focus on 184A1 nuclei treated with the histone deacetylase inhibitor TSA. At a Leiden resolution of 1.2, direct pixel clustering across all data identifies 20 pixel clusters (Extended Data Fig. 6a). In TSA-treated cells, clusters 10 and 19 are strongly increased in abundance, and clusters 2, 3, 4, 5, 7, 8, 11, 14, 16 are reduced in abundance (Extended Data Fig. 6b). To extract biological insights from this data, one must examine all of these clusters to identify what they correspond to, and then determine why they change in abundance in the clustering. Focusing on cluster 10 (which increases in abundance upon TSA-treatment), it can be seen that this is strongly enriched for histone H3 lysine 27 acetylation (H3K27ac), however there are also numerous other channels enriched in cluster 10, such as POLR2A-S2P, NONO and CDK9. Plotting clusters on a few example cells, we see that cluster 10 represents a specific subset of nucleoplasmic pixels that predominantly occur in TSA-treated cells (Extended Data Fig. 6c). This enrichment for a pixel cluster that is defined by high H3K27ac is expected because TSA treatment results in increased global histone acetylation. Using direct pixel clustering, however, it is difficult to obtain detailed insights into whether TSA has other effects on subcellular organisation because changes in H3K27ac result in reassignment of a large fraction of nuclear pixels to different clusters. For example, how should the reductions in abundance of clusters 2, 3, 4, 5, 7, 8, 11,14,16 be interpreted? Using CAMPA to analyse the same data, we instead learn a common set of seven nuclear CSLs across all conditions (Extended Data Fig. 6d), and can then examine how each 4i marker intensity changes within these identified

CSLs (Extended Data Fig. 6e). Doing so reveals that there is very little change to the size or the molecular composition of any of the CSLs, with the exception of increased H3K27ac that occurs uniformly across all nuclear subcompartments (Extended Data Fig. 6e). CAMPA therefore greatly simplifies the analysis of this perturbation condition compared to the direct pixel clustering approach.

An alternative method to ‘save’ the direct pixel clustering approach in this case would be to omit H3K27ac from the clustering because it varies globally between conditions, and thereby may allow identification of consistent clusters across conditions in TSA-treated and unperturbed cells. However, this would then require any measured protein that varies in intensity between conditions to be omitted from the clustering, which would rapidly limit the scalability to a large number of conditions. For example, at least eight of the 43 channels vary significantly in at least one of the six perturbations studied here (Fig. 1d), and at least two more vary across different cell-cycle stages (Extended Data Fig. 3a). Moreover, if the omitted channels include structural markers, then this approach may fail to recognize certain important subcellular landmarks. By instead using a conditional model, CAMPA removes this requirement to choose input channels *a priori* and allows the model to learn which proteins change in intensity between conditions during model training.

Supplementary Table 1: Antibodies and other reagents.

REAGENT	SOURCE	IDENTIFIER
Antibodies		
Mouse monoclonal anti-ALYREF (1:400)	Santa Cruz Biotechnology	Cat#sc-32311; Clone#11G5; Lot#G1517; RRID: AB_626667
Rabbit polyclonal anti-CALR (1:500)	Abcam	Cat#ab2907; Lot#GR3206421-2; RRID: AB_303402
Mouse monoclonal anti-CCNT1 (1:150)	Santa Cruz Biotechnology	Cat#sc-271348; Clone#E-3; Lot#J2617; RRID: AB_10608086
Mouse monoclonal anti-CDK7 (1:150)	Santa Cruz Biotechnology	Cat#sc-7344; Clone#C-4; Lot#L011; RRID: AB_627243
Rabbit monoclonal anti-CDK9 (1:400)	Cell Signaling Technology	Cat#2316; Clone#C12F7; Lot#7; RRID: AB_2291505
Mouse monoclonal anti-COIL (1:800)	Abcam	Cat#ab87913; Clone#IH10; Lot#GR50257-2; RRID: AB_10860831
Mouse monoclonal anti-CTNNB1 (1:200)	Cell Signaling Technology	Cat#2677; Clone#L54E2; Lot#3; RRID: AB_1030943
Rabbit polyclonal anti-DDX6 (1:800)	Bethyl Laboratories	Cat#A300-461A; RRID: AB_2277216
Mouse monoclonal anti-GOLGA2 (GM130) (1:300)	BD Biosciences	Cat#610823; Lot#7163670; RRID:AB_398142
Rabbit polyclonal anti-GTF2B (1:500)	Atlas Antibodies	Cat#HPA061626; Lot#R86237; RRID:AB_2684570
Chicken polyclonal anti-H2B (1:1000)	Abcam	Cat#ab134211; RRID: AB_2889048

Rabbit polyclonal anti-H3 (1:2500)	Abcam	Cat#ab1791; Lot#GR3190126-1; RRID: AB_302613
Rabbit polyclonal anti-H3K27ac (1:2500)	Abcam	Cat#ab4729; Lot#GR3231937-1; RRID: AB_2118291
Rabbit polyclonal anti-H3K4me3 (1:1200)	Abcam	Cat#ab8580; Lot#GR3190162-1; RRID: AB_306649
Rabbit polyclonal anti-HDAC3 (1:500)	Abcam	Cat#ab7030; Lot#GR166225-20; RRID: AB_305708
Mouse monoclonal anti-HSPD1 (HSP60) (1:8000)	Abcam	Cat#ab13532; Clone#Mab11-13; Lot#GR197160-6; RRID: AB_300433
Rabbit polyclonal anti-KPNA1 (1:300)	Laboratory of Karsten Weis[2]	RRID: N/A
Rabbit polyclonal anti-KPNA2 (1:500)	Laboratory of Karsten Weis[2]	RRID: N/A
Rabbit polyclonal anti-NCL (C23) (1:1000)	Santa Cruz Biotechnology	Cat#sc-13057; Lot#G3114; RRID: AB_2229696
Mouse monoclonal anti-NONO (1:2000)	Laboratory of Archa Fox[3]	RRID: N/A
Mouse monoclonal anti-PABPC1 (1:150)	Santa Cruz Biotechnology	Cat#sc-32318; Clone#10E10; Lot#D1913; RRID: AB_628097
Rabbit monoclonal anti-PABPN1 (1:250)	Abcam	Cat#ab75855; Clone#EP3000Y; Lot#GR3202568-6; RRID: AB_1310538
Rabbit polyclonal anti-pCDK9 (pThr186) (1:125)	Cell Signaling Technology	Cat#2549; Lot#2; RRID: AB_2077300
Rabbit monoclonal anti-PCNA (1:1000)	Cell Signaling Technology	Cat#13110; Clone#D3H8P; Lot#4; RRID: AB_2636979

Rabbit monoclonal anti-pMAPK1 (ERK-pThr202/204) (1:200)	Cell Signaling Technology	Cat#4370; Clone# D13.14.4E; Lot#24; RRID: AB_2315112
Mouse monoclonal anti-PML (1:75)	Santa Cruz Biotechnology	Cat#sc-966; Clone#PG-M3; Lot#K1215; RRID: AB_628162
Mouse monoclonal anti-POLR2A (RPB1) (1:200)	Santa Cruz Biotechnology	Cat#sc-55492; Clone#F-12; Lot#C0119; RRID: AB_630203
Rat monoclonal anti-POLR2A-S2P (1:500)	Millipore	Cat#04-1571; Clone#3E10; Lot#3169853; RRID: AB_10627998
Rat monoclonal anti-POLR2A-S5P (1:1500)	Millipore	Cat#04-1572; Clone#3E8; RRID:AB_10615822
Rabbit monoclonal anti-pRB1 (pSer807/811) (1:300)	Cell Signaling Technology	Cat#8516; Clone#D20B12; Lot#8; RRID: AB_11178658
Rabbit monoclonal anti-pRPS6 (pSer235/236) (1:300)	Cell Signaling Technology	Cat#4858; Clone#D57.2.2E; Lot#16; RRID: AB_916156
Mouse monoclonal anti-PXN (1:500)	BD Biosciences	Cat# 610052; RRID: AB_397464
Mouse monoclonal anti-RPS6 (1:175)	Cell Signaling Technology	Cat#2317; Clone#54D2; Lot#4; RRID: AB_2238583
Rabbit polyclonal anti-SETD1A (1:100)	Atlas Antibodies	Cat#HPA020646; Lot#A96712; RRID:AB_1856752
Mouse monoclonal anti-Sm (1:500)	David Spector[4]	Clone#Y12; RRID: AB_2692320
Rabbit polyclonal anti-SON (1:1500)	Atlas Antibodies	Cat#HPA023535; RRID: AB_1857362
Rabbit polyclonal anti-SP100 (1:800)	Atlas Antibodies	Cat#HPA017384; Lot#A106767; RRID: AB_1857399

Rabbit polyclonal anti-SRRM2 (1:1000)	Atlas Antibodies	Cat#HPA041411; Lot#A90041; RRID: AB_10796671
Mouse monoclonal anti-SRSF2 (1:1200)	Sigma-Aldrich	Cat#S4045; RRID:AB_477511
Mouse monoclonal anti-TUBA1A (1:3000)	Abcam	Cat#ab7291; Clone#DM1A; Lot#GR50257-2; RRID: AB_2241126
Mouse monoclonal anti-U2SNRNPB (B'') (1:400)	Laboratory of David Spector[5]	Clone#4G3; RRID: N/A
Mouse monoclonal anti-YAP1 (1:600)	Santa Cruz Biotechnology	Cat#sc-101199; Clone#63.7; Lot#K0513; RRID:AB_1131430
Goat polyclonal anti-Mouse IgG (H&L), Alexa Fluor 488 (1:500)	Thermo-Fisher	Cat#A11029; RRID: AB_138404
Goat polyclonal anti-Rabbit IgG (H&L), Alexa Fluor 568 (1:500)	Thermo-Fisher	Cat#A11036; RRID: AB_10563566
Goat polyclonal anti-Chicken IgY (H&L), Alexa Fluor 405 (1:1000)	Abcam	Cat#ab175674; RRID N/A
Goat polyclonal anti-Rabbit IgG (H&L), Alexa Fluor 488 (1:500)	Thermo-Fisher	Cat#A11034; RRID: AB_2576217
Goat polyclonal anti-Mouse IgG (H&L), Alexa Fluor 568 (1:500)	Thermo-Fisher	Cat#A11031; RRID: AB_144696
Goat polyclonal anti-Rat IgG (H&L), Alexa Fluor 568 (1:500)	Thermo-Fisher	Cat#A11077; RRID: AB_141874
Chemicals, Peptides, and Recombinant Proteins		
Alexa Fluor 647 Azide	Thermo-Fisher	Cat#A10277

Alexa Fluor 647 NHS Ester	Thermo-Fisher	Cat#A20006
Sodium ascorbate	Sigma-Aldrich	Cat#A7631
Copper sulfate	Sigma-Aldrich	Cat#7758-98-7
5-ethynyl uridine (5-EU)	Baseclick Gmbh	Cat#BCN-003
DAPI (4',6-diamidino-2-phenylindole, dihydrochloride)	Thermo-Fisher	Cat#D1306
Meayamycin	[6]	N/A
Triptolide	Adipogen Life Sciences	Cat#AG-CN2-0448
AZD4573	Selleckchem	Cat#S8719
CX5461	Axon Medchem	Cat#2173
Trichostatin A (TSA)	Selleckchem	Cat#S1045
Epidermal growth factor	Sigma-Aldrich	Cat#01-107
Insulin	Sigma-Aldrich	Cat#I1882
Hydrocortisone	Sigma-Aldrich	Cat#H0888

Cholera toxin	Sigma-Aldrich	Cat#C8052
Fibronectin	Sigma-Aldrich	Cat#F0895
Fetal bovine serum	Sigma-Aldrich	Cat#F7524
Horse serum	Gibco	Cat#16050122
DMEM, high glucose	GIbco	Cat#41965062
DMEM/F12	Gibco	Cat#11330032
OptiMEM Reduced Serum Medium	Gibco	Cat# 31985070
Penicillin/Streptomycin	Gibco	Cat#15140122
Lipofectamine RNAimax transfection reagent	Invitrogen	Cat#13778150
16% Paraformaldehyde	Electron Microscopy Sciences	Cat#EMS-15710
Intercept blocking buffer	Li-Cor	Cat#927-70001
Triton X-100	Sigma-Aldrich	Cat#T8787
Experimental Models: Cell Lines		

Human: HeLa, cervical cancer cell line (single-cell clone)	[7]	Kyoto
Human: 184A1, breast epithelial cell line (single-cell clone)	This paper	ATCC CRL-8798
Oligonucleotides		
Negative control siRNA #1; Ambion Silencer Select	Thermo-Fisher	Cat#4390843
siRNAs targeting *SBF2*; Ambion Silencer Select	Thermo-Fisher	Cat#s37818, s37819, s37820
Other		
384-well µClear plates	Greiner Bio-One	Cat#781092

Supplementary Table 2: Number of 184A1 cells removed during data cleanup across all conditions.

	n	% total
**All (non-border) cells imaged**	**13053**	**100%**
Mitotic cells (excluded)	636	4.9%
Polynucleated cells (excluded)	343	2.6%
Extreme DNA content (excluded)	92	0.7%
Acquisition error (excluded)	103	0.8%
Background artefacts (excluded)	31	0.2%
**Final analysed cells**	**11848**	**90.8%**

Supplementary Table 3: Number of 184A1 cells and pixels analysed per condition. Untreated, DMSO (2.5h) treated, and DMSO (12h) treated cells are referred to as ‘unperturbed’ (see Methods).

Condition	Number of cells	Number of wells	Number of pixels (nuclei)	Mean area (nuclei)
AZD4573 (2.5h)	1186	2	1.7E+07	1.4E+04
AZD4573 (1h)	1942	3	2.6E+07	1.4E+04
CX5461 (2.5h)	1552	3	2.0E+07	1.3E+04
DMSO (2.5h)	528	1	6.7E+06	1.3E+04
DMSO (12h)	606	1	7.7E+06	1.3E+04
Meayamycin (12h)	755	2	1.2E+07	1.6E+04
Untreated	2546	4	3.3E+07	1.3E+04
Triptolide (2.5h)	1018	2	1.3E+07	1.2E+04
TSA (1h)	1715	3	2.2E+07	1.3E+04
**Total**	**11848**	**21**	**1.6E+08**	

Supplementary Table 4: Number of HeLa cells removed during data cleanup across all conditions.

	**n**	**% total**
**All (non-border) cells imaged**	**3161**	**100%**
Mitotic cells (excluded)	193	6.1%
Polynucleated cells (excluded)	80	2.5%
Extreme DNA content (excluded)	101	3.2%
Acquisition error (excluded)	43	1.4%
Background artefacts (excluded)	10	0.3%
**Final analysed cells**	**2731**	**86.4%**

Supplementary Table 5: Number of HeLa cells and pixels analysed per condition.

Condition	Number of cells	Number of wells	Number of pixels (cell)	Mean area (cell)
Scrambled siRNA	2301	3	1.8E+08	7.8E+04
SBF2 siRNA	430	3	9.1E+07	2.1E+05
**Total**	**2731**	**6**	**2.7E+08**	

Supplementary Table 6: Number of cells and molecular profiles per split used for dataset training.

	train	validation	test
**184A1 dataset**			
cells	9’472	1’181	1’195
subsampled molecular profiles	615’240	76’432	76’956
**HeLa dataset**			
cells	2’160	284	287
subsampled molecular profiles	10’628’809	1’394’437	1’390’160

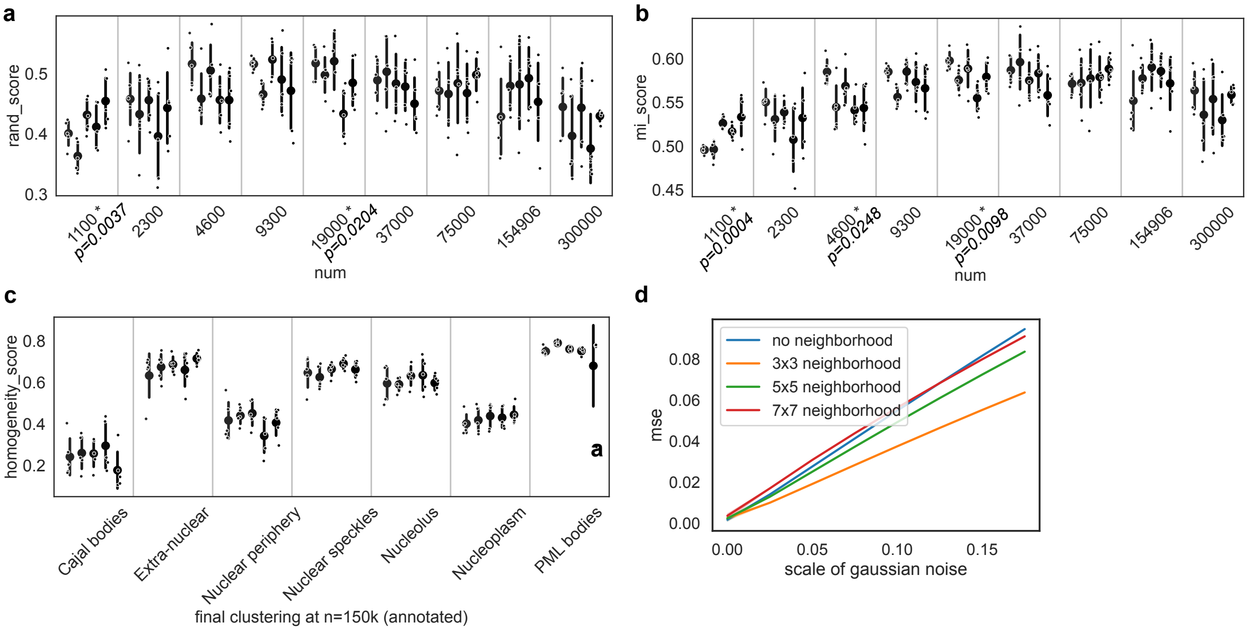

Supplementary Figure 1: Robustness to noise and randomness in subsampling and clustering using the 184A1 dataset.

a,b: Cluster stability at different subsample sizes. For each subsample size, clusterings from 5 different subsamples and 5 different Leiden initialisations in each subsample are computed and compared to the final CSLs obtained at 150k samples. Cluster overlap is assessed with adjusted Rand index (a) and adjusted mutual information (b). Scores are grouped by subsample; bars, 95% confidence intervals obtained by bootstrapping; centre dot, mean; points, individual data points. An asterisk * at a subsample size indicates a significant ($p<0.05$) effect of the subsample on the score, obtained by ANOVA. Different subsamples have no significant influence on cluster stability for subsamples sizes >= 37k.

c: Homogeneity of different clusterings at 150k samples with respect to final CSLs. Scores are grouped by subsample; bars, 95% confidence intervals obtained by bootstrapping; centre dot, mean; points, individual data points. Homogeneity is high for a certain CSL if, for each cluster in a clustering, all points assigned to this cluster either belong or not belong to the CSL. Homogeneity was lowest for Nucleoplasm, Nuclear periphery, and Cajal bodies, showing that the boundaries between these clusters are not as clearly defined from the data as for Nuclear Speckles, PML bodies, and Nucleolus CSLs.

d: Robustness of cVAE latent space when adding Gaussian noise to the input. Scale is the standard deviation of the Gaussian distribution. Shown is the Mean-Squared Error (MSE) and 95% confidence interval of pairwise comparisons with 5 different random seeds. cVAE latent spaces obtained by training with a local 1x1 (no neighbours), 3x3, 5x5, and 7x7 neighbourhood as context for every molecular profile are compared. Using a neighbourhood of 3x3 adds to increased stability of the latent space in the presence of single pixel noise. Using larger neighbourhoods reduces those benefits, as the model is more dependent upon specific configurations of neighbouring pixels for the generation of the latent space representation.

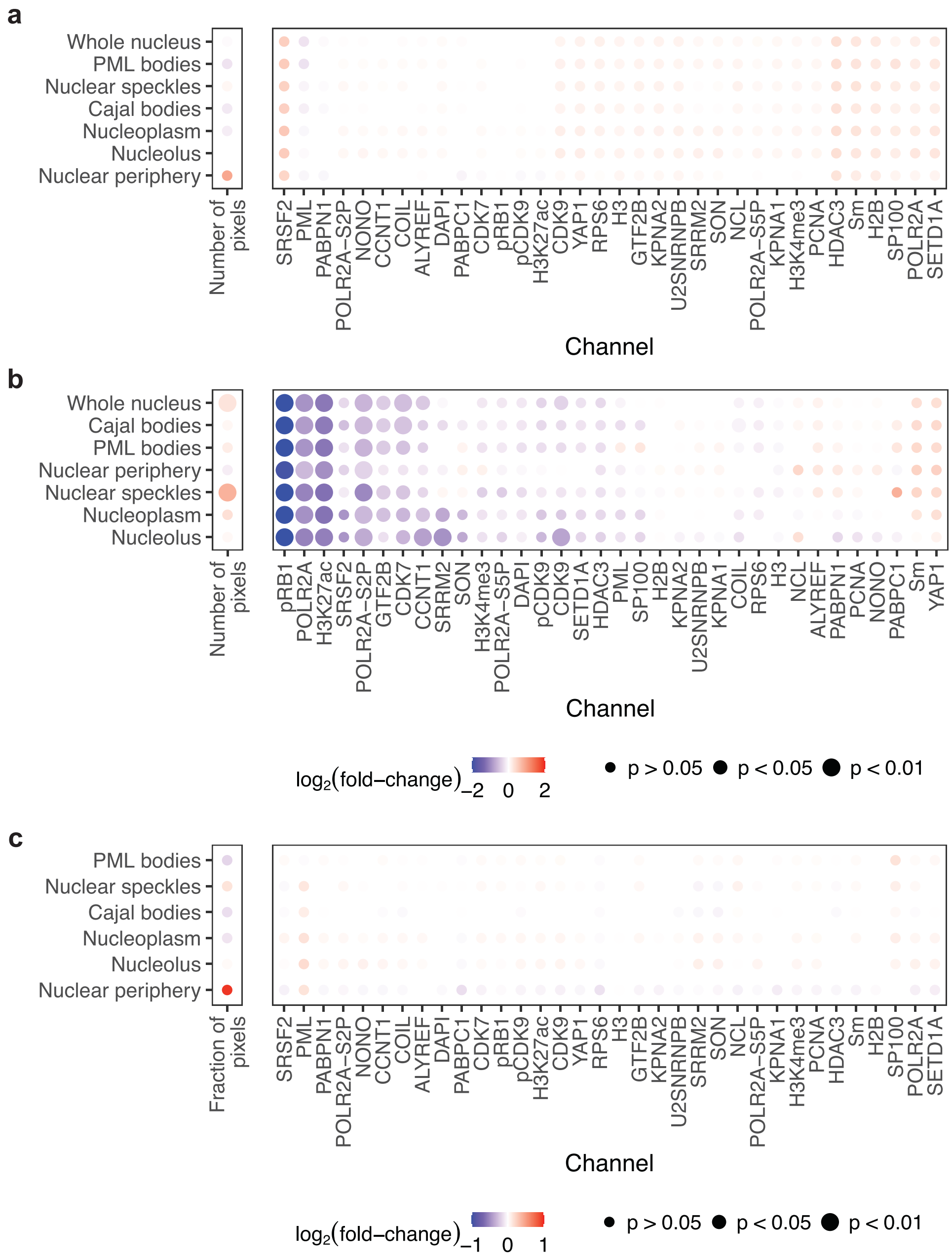

Supplementary Figure 2: Mean intensity changes upon Meayamycin or DMSO treatment.
a: Log2 fold-change of mean intensities for each channel in each CSL, or number of pixels in each CSL, when comparing DMSO with untreated cells. P-values show significance of DMSO treatment on channel intensities for each channel/CSL, as determined from the mixed effect model (Wald test, multiple testing correction using Benjamini-Yekutieli method). DMSO and untreated cells show similar mean intensities across all CSLs and are pooled as ‘unperturbed’ cells in subsequent analyses.
b: As in a, comparing Meayamycin treated cells with unperturbed cells.
c: As in a, comparing DMSO with untreated cells, except normalised by the overall (whole-nucleus) changes in intensity. In this case p-values indicate significance of mean intensity change in CSL compared to the change observed for the whole nucleus (see Methods).

a

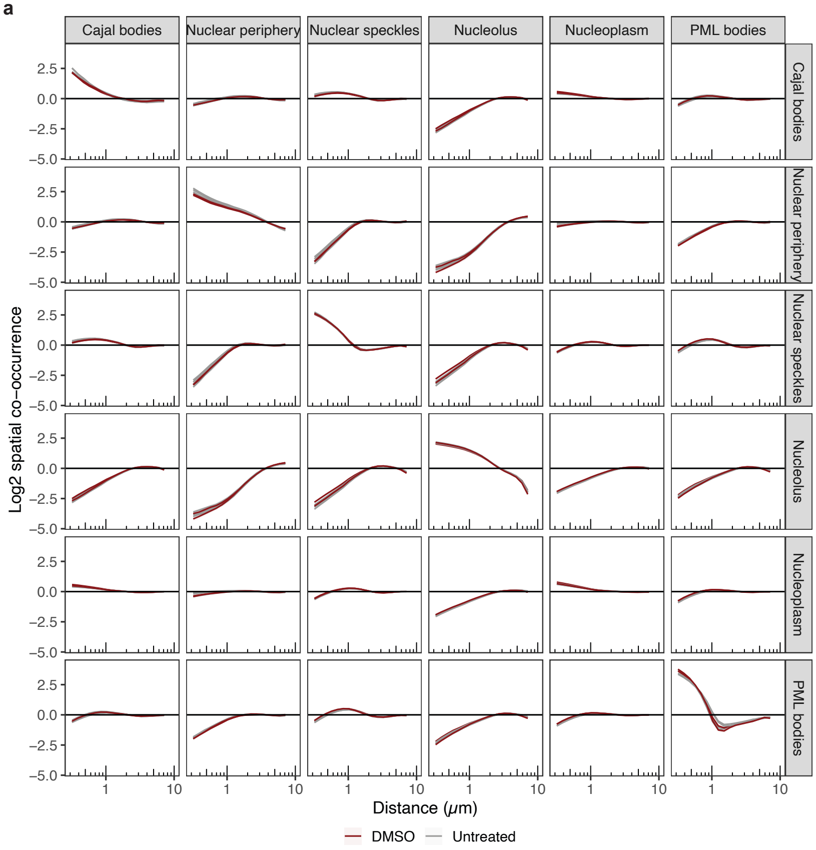
Cajal bodies
Nuclear periphery
Nuclear speckles
Nucleolus
Nucleoplasm
PML bodies
Log2 spatial co−occurrence
2.5
0.0
−2.5
−5.0
1
10
Distance (μm)
DMSO
Untreated

b

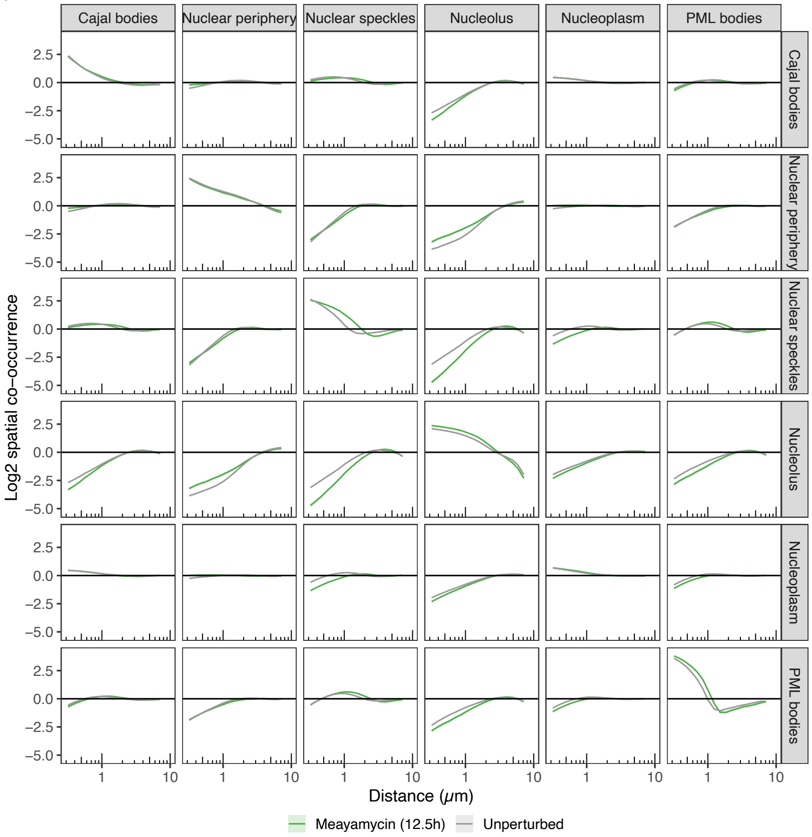
Cajal bodies
Nuclear periphery
Nuclear speckles
Nucleolus
Nucleoplasm
PML bodies
Log2 spatial co−occurrence
2.5
0.0
−2.5
−5.0
1
10
Distance (μm)
Meayamycin (12.5h)
Unperturbed

Supplementary Figure 3: Pairwise co-occurrence scores between CSLs.
a: Pairwise mean log2 spatial co-occurrence between all CSLs as a function of distance on x-axis (minimum of distance interval; on log scale) for DMSO and untreated control wells (pooled as ‘Unperturbed’ in Fig 3g). Each well plotted as an individual line. Shaded regions indicate 95% confidence interval for the mean. DMSO and untreated cells show similar co-occurrence scores and are pooled as ‘unperturbed’ cells in subsequent analyses.
b: Pairwise mean log2 spatial co-occurrence between all CSLs as a function of distance on x-axis (on log scale) for Meayamycin-treated and unperturbed cells. Shaded regions indicate 95% confidence intervals for the mean.

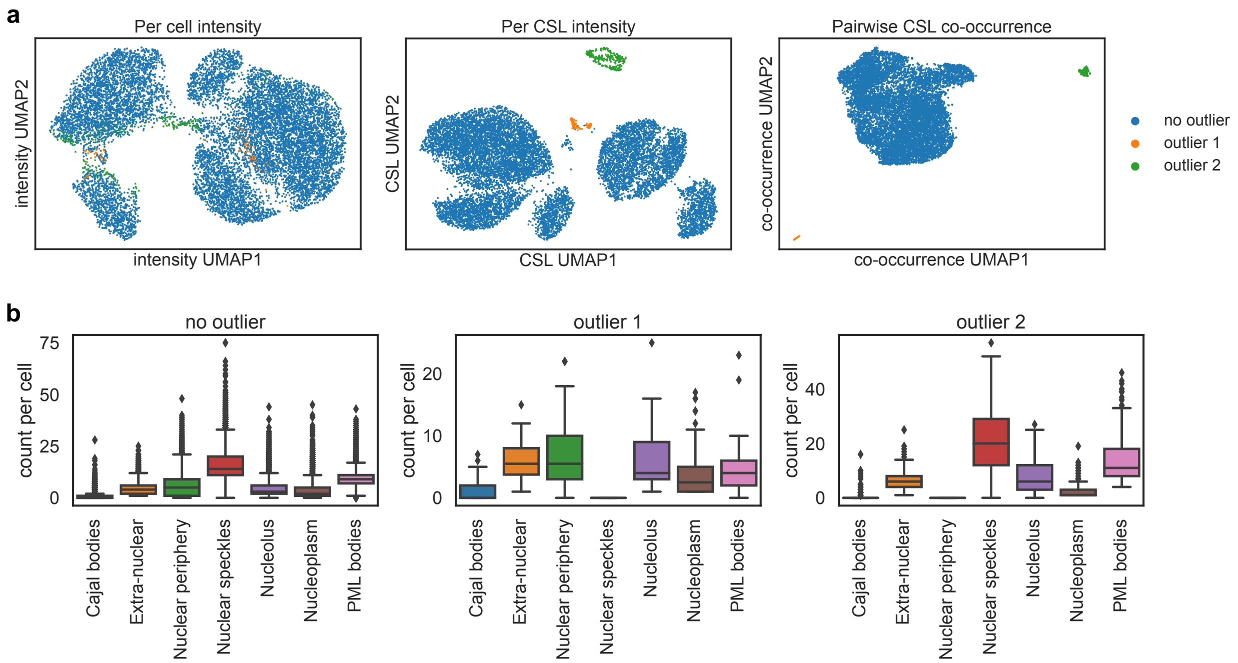

Supplementary Figure 4: Examination of outlier cells excluded in Fig. 4 on the basis of the co-occurrence UMAP.

Two outlier clusters were identified (outlier 1: 88 cells, outlier 2: 256 cells).

a: UMAP embedding of cells using different cellular features. Points colored by outlier clusters. The identified outlier clusters are visible outliers on the intensity UMAP and the co-occurrence UMAP.

b: Barplot of number of individual CSLs per cell, colored by CSL, for non-outlier cells (n=11504), and the two outlier clusters. Cells in outlier cluster 1 do not contain the Nuclear speckle CSL, and cells in outlier cluster 2 do not contain the Nuclear periphery CSL. Center line, median; box limits, upper and lower quartiles; whiskers, 1.5x interquartile range; points, outliers.

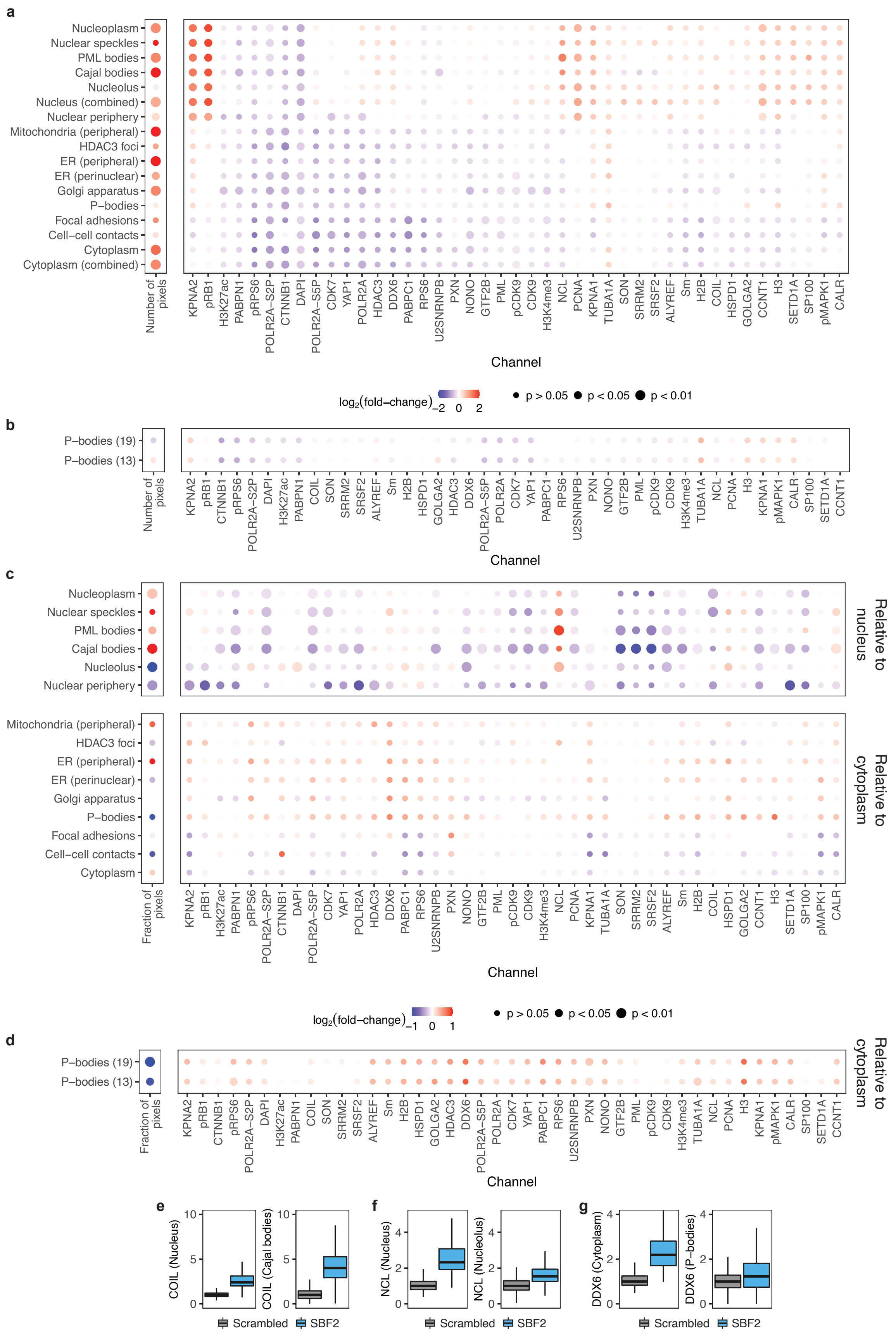
a
Nucleoplasm
Nuclear speckles
PML bodies
Cajal bodies
Nucleolus
Nucleus (combined)
Nuclear periphery
Mitochondria (peripheral)
HDAC3 foci
ER (peripheral)
ER (perinuclear)
Golgi apparatus
P–bodies
Focal adhesions
Cell–cell contacts
Cytoplasm
Cytoplasm (combined)
Number of pixels
KPNA2 pRB1 H3K27ac PABPN1 pRPS6 POLR2A–S2P CTNNB1 DAPI POLR2A–S5P CDK7 YAP1 POLR2A HDAC3 DDX6 PABPC1 RPS6 U2SNRNPB PXN NONO GTF2B PML pCDK9 CDK9 H3K4me3 NCL PCNA KPNA1 TUBA1A SON SRRM2 SRSF2 ALYREF Sm H2B COIL HSPD1 GOLGA2 CCNT1 H3 SETD1A SP100 pMAPK1 CALR
Channel
log2(fold–change) –2 0 2
p > 0.05 p < 0.05 p < 0.01
b
P–bodies (19)
P–bodies (13)
Number of pixels
KPNA2 pRB1 CTNNB1 pRPS6 POLR2A–S2P DAPI H3K27ac PABPN1 COIL SON SRRM2 SRSF2 ALYREF Sm H2B HSPD1 GOLGA2 HDAC3 DDX6 POLR2A–S5P POLR2A CDK7 YAP1 PABPC1 RPS6 U2SNRNPB PXN NONO GTF2B PML pCDK9 CDK9 H3K4me3 TUBA1A NCL PCNA H3 KPNA1 pMAPK1 CALR SP100 SETD1A CCNT1
Channel
c
Nucleoplasm
Nuclear speckles
PML bodies
Cajal bodies
Nucleolus
Nuclear periphery
Relative to nucleus
Mitochondria (peripheral)
HDAC3 foci
ER (peripheral)
ER (perinuclear)
Golgi apparatus
P–bodies
Focal adhesions
Cell–cell contacts
Cytoplasm
Relative to cytoplasm
Fraction of pixels
KPNA2 pRB1 H3K27ac PABPN1 pRPS6 POLR2A–S2P CTNNB1 DAPI POLR2A–S5P CDK7 YAP1 POLR2A HDAC3 DDX6 PABPC1 RPS6 U2SNRNPB PXN NONO GTF2B PML pCDK9 CDK9 H3K4me3 NCL PCNA KPNA1 TUBA1A SON SRRM2 SRSF2 ALYREF Sm H2B COIL HSPD1 GOLGA2 CCNT1 H3 SETD1A SP100 pMAPK1 CALR
Channel
log2(fold–change) –1 0 1
p > 0.05 p < 0.05 p < 0.01
d
P–bodies (19)
P–bodies (13)
Relative to cytoplasm
Fraction of pixels
KPNA2 pRB1 CTNNB1 pRPS6 POLR2A–S2P DAPI H3K27ac PABPN1 COIL SON SRRM2 SRSF2 ALYREF Sm H2B HSPD1 GOLGA2 HDAC3 DDX6 POLR2A–S5P POLR2A CDK7 YAP1 PABPC1 RPS6 U2SNRNPB PXN NONO GTF2B PML pCDK9 CDK9 H3K4me3 TUBA1A NCL PCNA H3 KPNA1 pMAPK1 CALR SP100 SETD1A CCNT1
Channel
e
COIL (Nucleus)
COIL (Cajal bodies)
f
NCL (Nucleus)
NCL (Nucleolus)
g
DDX6 (Cytoplasm)
DDX6 (P–bodies)
Scrambled SBF2

Supplementary Figure 5: Quantification of mean intensity changes of each channel in each CSL in HeLa cells upon *SBF2*-knockdown.

a: Log2 fold-change of mean intensities for each channel in each CSL, or number of pixels in each CSL, when comparing *SBF2*-knockdown with scrambled siRNA control cells. P-values show significance of *SBF2*-knockdown on channel intensities for each channel/CSL, as determined from the mixed effect model (Wald test, multiple testing correction using Benjamini-Yekutieli method).
b: As in a, for the non-merged CSLs corresponding to P-bodies.
c: As in a, except normalised by the whole nucleus or whole cytoplasm (not the “Cytoplasm” CSL) changes in intensity, respectively for the upper and lower panels. In this case p-values indicate significance of mean intensity change in CSL compared to the change observed for the nucleus or cytoplasm, respectively (see Methods).
d: As in c, for the non-merged CSLs corresponding to P-bodies.
e: Sum intensity of COIL in the nucleus or Cajal bodies of *SBF2*-knockdown and scrambled siRNA control HeLa cells. Boxplots summarise distributions across cells. Center line, median; box limits, upper and lower quartiles; whiskers, 1.5x interquartile range; outliers omitted for clarity. Scrambled: n=2301; *SBF2*: n=430 (see Supplementary Table 5 for details).
f: As in c for NCL/Nucleolus.
g: As in c for DDX6/P-bodies.

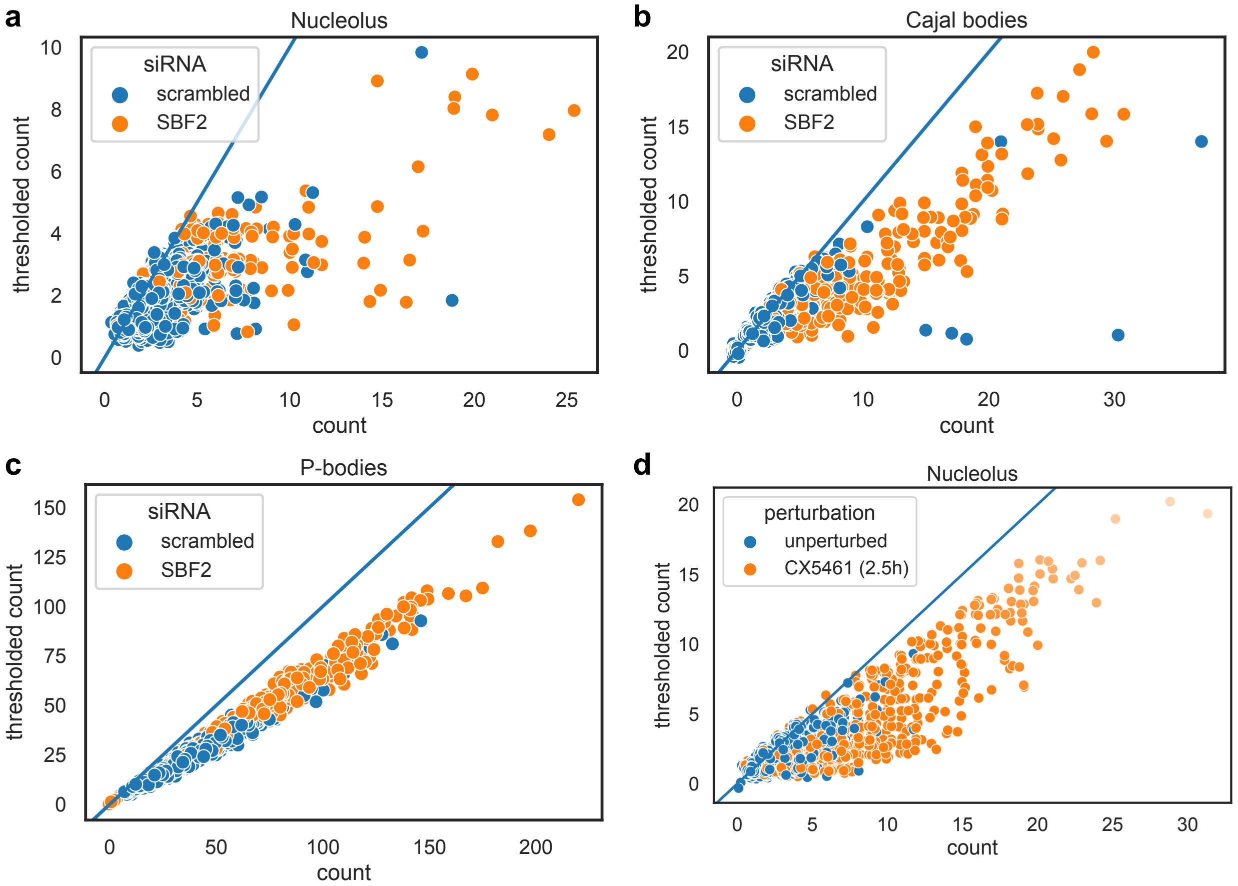

Supplementary Figure 6: Effect of size-filtering on CSL counts per cell.
Number of CSLs per cell before and after filtering small individual CSLs for different CSLs (<10px or cumulative sum of removed object <10% of total CSL area in this cell). From cells that contain more individual CSLs before filtering, more individual CSLs are removed, indicating that these cells contained many small individual CSLs. Blue line indicates diagonal. Small jitter (sigma=0.2) applied to each point for visualisation.

a: Nucleolus count in *SBF2*-knockdown and scrambled siRNA control HeLa cells.

b: Cajal body count in *SBF2*-knockdown and scrambled siRNA control HeLa cells.

c: P-body count in *SBF2*-knockdown and scrambled siRNA control HeLa cells.

d: Nucleolus count in unperturbed and CX5461-treated 184A1 cells.

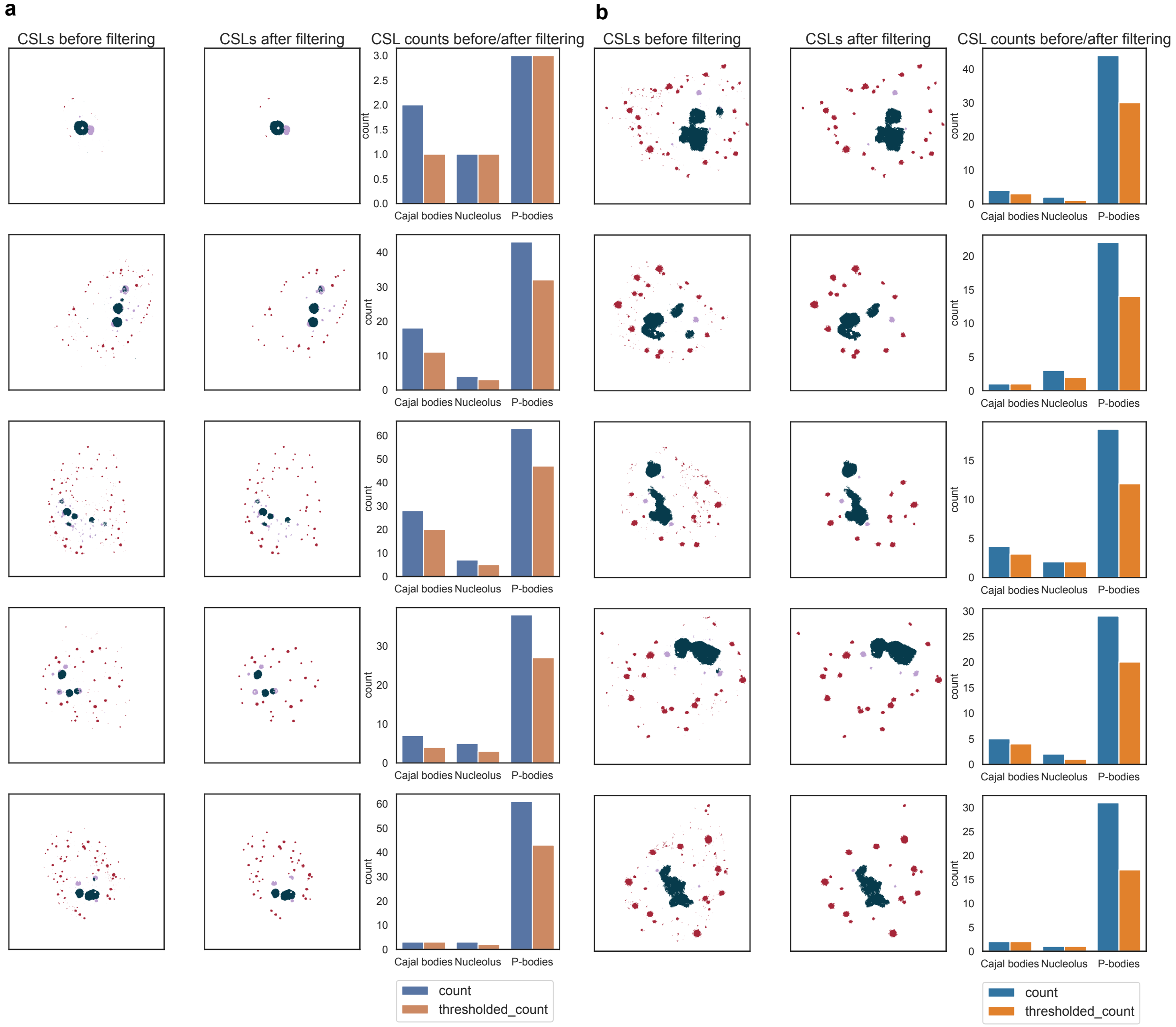

Supplementary Figure 7: Example cells showing CSLs before and after size-filtering. Cajal bodies, Nucleolus and P-bodies and their counts before and after filtering (which removes bodies <10px or those whose cumulative sum is <10% of total CSL area per cell) in HeLa cells (see Methods). Note that plot sizes differ between a and b (a: 700x700px, b: 400x400px).

a: Randomly selected cells from *SBF2*-knockdown condition. Left: Nucleolus (dark blue), Cajal body (purple), and P-body (red) CSLs before size-filtering. Middle: CSLs after size-filtering. Right: Barplot of CSL counts before and after filtering.

b: Same as in panel a, but for randomly selected cells from scrambled-siRNA condition.

References

1. Gut, G., Herrmann, M. D. & Pelkmans, L. Multiplexed protein maps link subcellular organization to cellular states. *Science* **361**, (2018).
2. Nachury, M. V., Ryder, U. W., Lamond, A. I. & Weis, K. Cloning and characterization of hSRP1γ, a tissue-specific nuclear transport factor. *Proceedings of the National Academy of Sciences* **95**, 582–587 (1998).
3. Lee, P. W. *et al.* Paraspeckle subnuclear bodies depend on dynamic heterodimerisation of DBHS RNA-binding proteins via their structured domains. *J. Biol. Chem.* **298**, 102563 (2022).
4. Pettersson, I., Hinterberger, M., Mimori, T., Gottlieb, E. & Steitz, J. A. The structure of mammalian small nuclear ribonucleoproteins. Identification of multiple protein components reactive with anti-(U1)ribonucleoprotein and anti-Sm autoantibodies. *J. Biol. Chem.* **259**, 5907–5914 (1984).
5. Habets, W., Hoet, M., Bringmann, P., Lührmann, R. & van Venrooij, W. Autoantibodies to ribonucleoprotein particles containing U2 small nuclear RNA. *EMBO J.* **4**, 1545–1550 (1985).
6. Albert, B. J. *et al.* Meayamycin inhibits pre-messenger RNA splicing and exhibits picomolar activity against multidrug-resistant cells. *Mol. Cancer Ther.* **8**, 2308–2318 (2009).
7. Battich, N., Stoeger, T. & Pelkmans, L. Control of Transcript Variability in Single Mammalian Cells. *Cell* **163**, 1596–1610 (2015).
