# Supplementary material for: Learning consistent subcellular landmarks to quantify changes in multiplexed protein maps
Source: Nat Methods. 2023 May 29;20(7):1058–69. doi: 10.1038/s41592-023-01894-z (PMC10333128; doi:10.1038/s41592-023-01894-z)
Supplement: Supplementary file 2 — Reporting Summary [file 41592_2023_1894_MOESM2_ESM.pdf]

nature portfolio

Corresponding author(s): Fabian Theis, Lucas Pelkmans

Last updated by author(s): Apr 5, 2023

Reporting Summary

Nature Portfolio wishes to improve the reproducibility of the work that we publish. This form provides structure for consistency and transparency in reporting. For further information on Nature Portfolio policies, see our Editorial Policies and the Editorial Policy Checklist.

Statistics

For all statistical analyses, confirm that the following items are present in the figure legend, table legend, main text, or Methods section.

n/a	Confirmed	
☐	☒	The exact sample size (*n*) for each experimental group/condition, given as a discrete number and unit of measurement
☐	☒	A statement on whether measurements were taken from distinct samples or whether the same sample was measured repeatedly
☐	☒	The statistical test(s) used AND whether they are one- or two-sided<br>*Only common tests should be described solely by name; describe more complex techniques in the Methods section.*
☒	☐	A description of all covariates tested
☐	☒	A description of any assumptions or corrections, such as tests of normality and adjustment for multiple comparisons
☐	☒	A full description of the statistical parameters including central tendency (e.g. means) or other basic estimates (e.g. regression coefficient) AND variation (e.g. standard deviation) or associated estimates of uncertainty (e.g. confidence intervals)
☐	☒	For null hypothesis testing, the test statistic (e.g. *F*, *t*, *r*) with confidence intervals, effect sizes, degrees of freedom and *P* value noted<br>*Give P values as exact values whenever suitable.*
☒	☐	For Bayesian analysis, information on the choice of priors and Markov chain Monte Carlo settings
☐	☒	For hierarchical and complex designs, identification of the appropriate level for tests and full reporting of outcomes
☐	☒	Estimates of effect sizes (e.g. Cohen's *d*, Pearson's *r*), indicating how they were calculated

Our web collection on statistics for biologists contains articles on many of the points above.

Software and code

Policy information about availability of computer code

Data collection	Data were acquired using an automated spinning-disk microscope (CellVoyager 7000, Yokogawa), using the proprietary CV7000 software (version R1.17.05).
Data analysis	Analysis was performed using CAMPA which is available at https://github.com/theislab/campa with docs at https://campa.readthedocs.io. All scripts necessary for reproducing the results and figures (except schematic figure panels Fig. 1a,b,c, Fig. 3f) can be found at https://github.com/theislab/campa_ana.<br>Nuclear and cell segmentation, identification of border cells, and cell-cycle classification was performed using TissueMAPS, an open-source project for high-throughput image analysis which is available at https://github.com/pelkmanslab/TissueMAPS. The TissueMaps analysis pipeline description with module files containing parameter settings used for the preprocessing of data in this paper is provided at https://github.com/theislab/campa_ana.<br>We used Ilastik (version 1.3.3) to train a model for nuclear and cell segmentation.<br>For statistical analysis of mean intensity and CSL abundance changes, we used the nlme package (version 3.1-153) in R version 3.6.3 to fit the mixed models and used emmeans (version 1.7.0) to extract estimates and perform hypothesis tests.

For manuscripts utilizing custom algorithms or software that are central to the research but not yet described in published literature, software must be made available to editors and reviewers. We strongly encourage code deposition in a community repository (e.g. GitHub). See the Nature Portfolio guidelines for submitting code & software for further information.

Data

Policy information about availability of data

All manuscripts must include a data availability statement. This statement should provide the following information, where applicable:

- Accession codes, unique identifiers, or web links for publicly available datasets
- A description of any restrictions on data availability
- For clinical datasets or third party data, please ensure that the statement adheres to our policy

The data used to generate all results and figures reported in this manuscript is available at https://doi.org/10.5281/zenodo.7299516. Pre-trained models and clusterings reported in the manuscript are available at https://doi.org/10.5281/zenodo.7299750.
CSL-derived features from the 184A1 and the HeLa datasets are available at https://doi.org/10.6084/m9.figshare.19699651.
The human protein atlas that was used to annotate CSLs is available at www.proteinatlas.org.

Human research participants

Policy information about studies involving human research participants and Sex and Gender in Research.

Reporting on sex and gender	N/A
Population characteristics	N/A
Recruitment	N/A
Ethics oversight	N/A

Note that full information on the approval of the study protocol must also be provided in the manuscript.

Field-specific reporting

Please select the one below that is the best fit for your research. If you are not sure, read the appropriate sections before making your selection.

☒ Life sciences ☐ Behavioural & social sciences ☐ Ecological, evolutionary & environmental sciences

For a reference copy of the document with all sections, see nature.com/documents/nr-reporting-summary-flat.pdf

Life sciences study design

All studies must disclose on these points even when the disclosure is negative.

Sample size	No sample-size calculation was performed. We sampled 1000-3000 cells per condition, across 2-4 replicate wells per condition (Supplementary Tables 3 and 5) based on experimental feasibility. This sample size enables statistical comparisons between conditions using mixed-effects models with random effects for each replicate well, as described in Methods.
Data exclusions	Mitotic and polynucleated cells were excluded during data cleanup, as described in Methods, and quantified in Supplementary Tables 2 and 4. In Figure 4a, a small subset of outlier cells were excluded from the UMAP plot. Justification of this exclusion is provided in Supplementary Figure 10, as indicated in Figure 4 legend. When analyzing CSL object features, small objects were removed as described in Methods, and indicated in Figure legends.
Replication	Data were from a single experiment. Replicate wells were included for all conditions.
Randomization	Plate layout was designed so that replicate wells were in different rows and columns of the plate, and were periodically interspersed with negative control wells with respect to image acquisition time.
Blinding	Investigators were not blinded during the experiments because we wanted to validate success of experimental perturbations (visually) before proceeding with several weeks of expensive immunofluorescence analysis. The analysis method is predominantly automated, from image acquisition to unsupervised training of machine learning models, to statistical analysis of quantitative data. An exception to this is the supervised training of machine-learning models as described in "Data exclusions".

Reporting for specific materials, systems and methods

We require information from authors about some types of materials, experimental systems and methods used in many studies. Here, indicate whether each material, system or method listed is relevant to your study. If you are not sure if a list item applies to your research, read the appropriate section before selecting a response.

Materials & experimental systems

n/a	Involved in the study
☐	☒ Antibodies
☐	☒ Eukaryotic cell lines
☒	☐ Palaeontology and archaeology
☒	☐ Animals and other organisms
☒	☐ Clinical data
☒	☐ Dual use research of concern

Methods

n/a	Involved in the study
☒	☐ ChIP-seq
☒	☐ Flow cytometry
☒	☐ MRI-based neuroimaging

Antibodies

Antibodies used	Refer to Supplementary Table 1
Validation	No additional validation undertaken for this study. Information for each can be found using the Research Resource Identifiers (RRIDs) in Supplementary Table 1

Eukaryotic cell lines

Policy information about cell lines and Sex and Gender in Research

Cell line source(s)	HeLa Kyoto (female) cell populations were derived from a single-cell clone (Battich et al., 2015).<br>184A1 ((ATCC CRL-8798; human female breast epithelial) cell populations were derived from a single-cell clone (Kramer et al., 2022).
Authentication	HeLa Kyoto (female) cell populations were tested for identity by karyotyping (Battich et al., 2015).<br>184A1 (human female breast epithelial) cell populations were not authenticated.
Mycoplasma contamination	All cells tested negative for mycoplasma contamination.
Commonly misidentified lines<br>(See ICLAC register)	No commonly misidentified lines were used
