# Supplementary figures and images for: Learning consistent subcellular landmarks to quantify changes in multiplexed protein maps
Source: Nat Methods. 2023 May 29;20(7):1058–69. doi: 10.1038/s41592-023-01894-z (PMC10333128; doi:10.1038/s41592-023-01894-z)

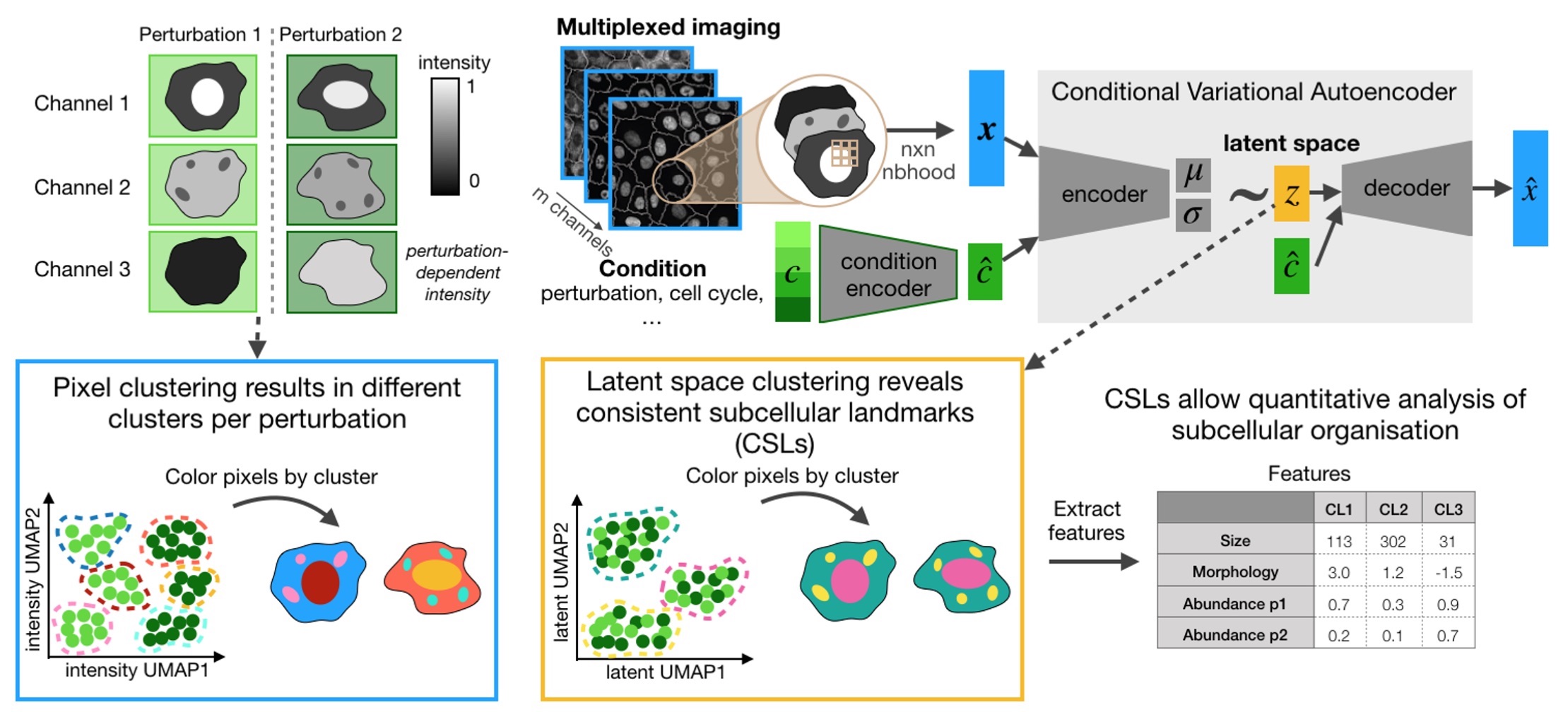

Supplement: Supplementary file 4 — CAMPA software [file 41592_2023_1894_MOESM4_ESM.zip › docs/source/_static/img/Figure1ab.jpg]
